# Ferroptotic Pathway Activation in Spermatogonia: A Novel Mechanism of Busulfan-Induced Testicular Injury

**DOI:** 10.3390/biology14060594

**Published:** 2025-05-23

**Authors:** Huanhuan Hu, Wenzheng Yuan, Yulin Wang, Zimei Dong, Guangwen Chen

**Affiliations:** 1 College of Life Science, Henan Normal University, Xinxiang 453007, China; huhuanhuan@sqmc.edu.cn (H.H.); 18348320739@163.com (Y.W.); 2 Key Laboratory of Fertility Preservation, School of Life Sciences and Technologies, North Henan Medical University, Xinxiang 453003, China; ywz1111261@163.com

**Keywords:** heme oxygenase 1, ferroptosis, busulfan, chemotherapy, oxidative stress, testis

## Abstract

Busulfan (BU) is a widely used chemotherapy drug that has been shown to cause reproductive functional impairment in humans and model animals. However, the precise mechanisms underlying testicular injury induced by BU exposure have not been fully elucidated. Ferroptosis is a form of programmed cell death mediated by iron-dependent lipid peroxidation. The aim of the current study was to determine whether ferroptosis was involved in BU-induced testicular injury. We demonstrated that exposure to BU led to an increase in iron content in the testes of mice. Subsequent western blotting and reverse transcription quantitative PCR, as well as staining of testicular tissue sections, confirmed that ferroptosis mediated BU-induced testicular injury. Consistent with our in vivo findings, we found that ferroptosis, including iron metabolism and the solute carrier family 7 member 11/glutathione peroxidase 4 (xCT/GPX4) signaling pathway, may play a key role in mediating BU-induced injury to GC-1 spg cells in vitro. Treatment with ferroptosis inhibitors slowed cell death caused by BU exposure. Specifically, we found that the administration of zinc protoporphyrin IX (ZnPP), a heme oxygenase 1 (HO1) inhibitor, rescued BU-induced cell death. In conclusion, our in vivo and in vitro findings confirmed that BU exposure led to testicular ferroptosis in mice via the iron intake pathway and the HO1 signaling pathway.

## Simple Summary

Busulfan, a chemotherapy drug with known reproductive toxicity, was investigated for its potential role in triggering testicular injury through ferroptosis, an iron-dependent cell death pathway. This study examined the effects of busulfan on testicular function, ferroptosis-related biomarkers (e.g., GPX4, lipid peroxidation), and spermatogenesis in both in vitro (germ cell lines) and in vivo (mouse model) systems. Results demonstrated that busulfan significantly activated the ferroptotic pathway, leading to mitochondrial dysfunction, reduced antioxidant defenses, and germ cell loss. While short-term interventions with ferroptosis inhibitors partially alleviated damage, long-term reproductive outcomes remained compromised. These findings highlight ferroptosis as a key mechanism underlying busulfan-induced testicular injury, suggesting targeted inhibition of this pathway as a potential strategy to mitigate chemotherapy-associated male infertility. Further studies are needed to optimize therapeutic approaches and assess clinical applicability.

## 1. Introduction

The global incidence of cancer continues to increase annually, and surgery combined with chemotherapy is the first line of treatment in most cases [1]. While chemotherapy has significantly increased cancer patients’ survival rates, its immediate and long-term side effects worry doctors, patients, and their families. Chemotherapy drugs can destroy both cancerous and non-cancerous cells, and some medications may impact male fertility [2,3,4]. Thus, the reproductive harm caused by chemotherapy drugs should not be overlooked [5].

Busulfan (BU), a cornerstone alkylating agent in chemotherapy and hematopoietic stem cell transplantation, is associated with dose-dependent long-term reproductive toxicity, particularly testicular injury, though its mechanistic underpinnings remain incompletely elucidated [6,7,8]. BU and its derivatives are thought to have anti-cancer properties by interfering with DNA synthesis, repair, and function, which in turn inhibits cell metabolism and stops cell growth, leading to the destruction of cancer cells [9]. Nevertheless, BU also affects the functions of healthy cells, tissues, and organs, including the male reproductive system [10,11,12]. The research showed that spermatogonia were the main target cell of male reproductive toxicity. The loss of spermatogonia leads to spermatogenesis disorders, and even oligospermia and azoospermia [13]. Based on these reports, BU is considered to be an ideal drug for establishing oligospermia and azoospermia in animal experimental models in order to better simulate clinical symptoms [14]. Elucidation of the molecular mechanisms underlying male reproductive toxicity not only provides a theoretical basis for the germ cell injury induced by BU, but also provides a potential target for clinical intervention in oligospermia and azoospermia.

Ferroptosis, a recently discovered form of non-apoptotic cell death, is triggered by iron accumulation and lipid peroxidation [15] and is distinct from other types of cell death. The main morphological features of ferroptosis include mitochondrial shrinkage with increased membrane density and reduced mitochondrial cristae. Biochemically, iron accumulation and increased reactive oxygen species (ROS) production from lipid peroxidation play crucial roles in triggering ferroptosis [16]. Ferroptosis is characterized by three key processes: (1) an increase in free iron content that generates ROS through the Fenton reaction; (2) depletion of the antioxidant Glutathione (GSH) and loss of Glutathione peroxidase 4 (GPX4) activity; and (3) accumulation of lipid oxidative damage, causing degeneration of cell membranes. The occurrence of each process can affect the sensitivity to ferroptosis [17]. Furthermore, increasing evidence has indicated that ferroptosis is closely associated with many biological processes, such as iron metabolism, amino acid metabolism, and lipid metabolism, which can affect the sensitivity of ferroptosis [18]. Ferroptosis has been implicated in multiple pathological processes including neurodegeneration, carcinogenesis, ulcerative colitis, and ischemia-reperfusion injury of the kidney and liver [19,20,21], highlighting the potential role of ferroptosis in the occurrence and development of various diseases.

Studies have shown that some compounds including iron chelator deferoxamine mesylate (DFO) and lipid peroxidation inhibitors ferrostatin-1 (Fer-1), GSH, and liproxstatin-1 are effective in preventing ferroptosis in male reproductive diseases [22]. However, whether ferroptosis is involved in BU-induced testicular injury remains unclear. BU exposure has recently been shown to lead to a significant increase in iron in the testes. In addition, Fer-1 has been shown to significantly alleviate intestinal mucositis caused by BU in vivo, suggesting that ferroptosis may be involved in BU-induced organic damage [23,24]. Therefore, the aim of the current study was to examine the effects and potential underlying mechanisms of action of BU in BU-induced male reproductive disease. Elucidating the ferroptotic pathway in BU-induced testicular injury may provide novel targets for adjuvant therapies (e.g., iron chelators or HO1 modulators) to preserve fertility during chemotherapy, aligning with the pharmaceutical development goals of precision medicine.

## 2. Materials and Methods

### 2.1. Animal Care and Experimental Design

Male C57BL/6J mice (20–22 g, 6–8 weeks) were obtained from Vital River Laboratory Animal Technology Co., Ltd. (Beijing, China). Mice were housed in a room with a 12 h/12 h light/dark cycle, and acclimatized to the room for 7 days prior to experiments. After 1 week of adaptive feeding, mice were randomly divided into two groups: (1) 0.9% NaCl injection group (n = 10), and (2) BU (Sigma, St. Louis, MO, USA, F809394) injection group (n = 10). The mice in the BU injection group were intraperitoneally injected with a single dose of BU (40 mg/kg). The BU dose gradient was determined from earlier research [25,26]. After 28 days, all mice were then sacrificed via cervical dislocation, and we immediately harvested their serum and testes. The samples were quick-frozen in liquid nitrogen and frozen for storage at −80 °C. All animal procedures were approved by the Institutional Animal Care and Use Committee (IACUC) at the Sanquan College of Xinxiang Medical University (SQ2023110701).

### 2.2. Testicular Function and Histology

The testicular organ index was calculated using the formula:

Organ Index (mg/g) = [Testicular Weight (mg)]/[Body Weight (g)]

This ratio normalizes organ weight to the animal’s body weight, eliminating individual size variations as a confounding factor.

Testicular function was assessed in mice using commercially available ELISA kits to measure their serum testosterone levels according to the manufacturer’s instructions (ELK Biotechnology, Wuhan, China).

The fresh tissues were fixed in 4% paraformaldehyde and embedded in paraffin. Testicular sections (5 μm) were stained with hematoxylin and eosin (H&E). Morphological examination was performed using a light microscope (TANON, Shanghai, China). The Johnson score was employed to quantitatively assess the status of spermatogenesis [27,28].

### 2.3. RNA Sequencing (RNA-Seq) Analysis

Mice were treated with BU. Twenty-eight days later, the testes were collected and subjected to RNA-seq analysis. RNA extraction, library construction, and data analysis were performed as previously described [29]. In summary, mRNA sequencing analyses were conducted on mouse testes, with each group having three replicates. We performed mRNA sequencing on an Illumina NovaSeq 6000 platform at LC Bio-Technology Company (Hangzhou, China). DESeq2 was used to select the differentially expressed genes (DEGs). Furthermore, Gene set enrichment analysis (GSEA), Gene Ontology (GO), and Kyoto Encyclopedia of Genes and Genomes (KEGG) enrichment analyses were performed for performing the functional analysis of the DEGs.

### 2.4. Terminal Deoxynucleotidyl Transferase dUTP Nick End-Labeling (TUNEL) Staining

Serial slices (10 μm-thick) were cut from frozen testes sections and subjected to TUNEL staining using a commercially available TUNEL Staining Kit from Solarbio (Beijing, China). Sections were washed with PBS after each step. Images were captured using a fluorescent microscope (Leica, Wetzlar, Germany).

### 2.5. Cell Lines and Viability Assay

The spermatogonial GC-1 spg cells, an immortalized cell line derived from an adult mouse testis, were obtained from the China Cell Culture Center (Shanghai, China). GC-1 spg cells were cultured in DMEM supplemented with 10% fetal bovine serum and antibiotics at 37 °C in a humidified atmosphere incubator consisting of 5% CO_2_ (Tokyo, Japan).

GC-1spg cells were seeded onto 96-well plates at a density of 5000 cells per well, then treated with various drugs as described below. Cell viability was assessed using a Cell Counting Kit-8 (CCK8, Glpbio, Montclair, CA, USA, GK10001) and YO-PRO-1/PI (Beyotime, Shanghai, China, C1075) according to the manufacturer’s instructions.

### 2.6. Transmission Electron Microscopy

GC-1 spg cells that were treated were collected, postfixed with 1% osmium tetroxide, dehydrated using a series of ethanol gradients, and then embedded in epoxy resin. Ultrathin sections (50 nm) were cut, stained with uranyl acetate and lead citrate, and visualized using a Hitachi microscope (Tokyo, Japan).

### 2.7. Treatment with Ferroptosis and Heme Oxygenase 1 (HO1) Inhibitors

Before treatment with BU, cells were pretreated with 1 μM ferroptosis-specific inhibitor ferrostatin-1 (Glpbio, GC10380) and 50 μM deferoxamine mesylate (Glpbio, GC13554), or 1 μM HO1 inhibitor zinc protoporphyrin IX (ZnPP, Glpbio, GC11208), or 5 mM oxidative stress inhibitor N-acetylcysteine (NAC, Sigma, A9165) and GSH (Sigma, G6529) for 1 h.

### 2.8. Measurement of Iron, GSH, and Malondialdehyde (MDA) Content

Total cellular iron and non-heme iron levels in the testes tissue samples were measured using commercially available colorimetric assay kits according to the manufacturer’s instructions (Nanjing Jiancheng Bioengineering Institute, Nanjing, China, A039-2). Similarly, GSH and MDA levels in testes tissue samples and cell lysates were measured using commercially available colorimetric assay kits according to the manufacturer’s instructions (Nanjing Jiancheng Bioengineering Institute, A006-2 and A003-1).

### 2.9. Reverse Transcription Quantitative PCR (RT-qPCR) and Western Blotting

Total RNA was extracted from tissues and cells using TRIzol reagent (Invitrogen, Carlsbad, CA, USA), then reverse transcribed into cDNA using the Reverse Transcription Kit (Takara, Tokyo, Japan). The RT-qPCR was carried out as previously described. The 2^−ΔΔCt^ formula was used to compare the relative gene expression levels, which were normalized to the housekeeping gene GAPDH. Information about the primers used in this study can be found in the Supplementary File (Table S1).

Western blotting was performed as described using primary antibodies against cystine/glutamate antiporter solute carrier family 7 member 11 (xCT/SLC7A11, CST, 98051), ferritin (FTH, CST, 4393), GPX4 (CST, 52455S), nuclear factor erythroid 2-related factor 2 transferrin receptor (NRF2, CST, 12721S), (TFR, Abcam, Cambridge, MA, USA, ab214039), transferrin (TF, Proteintech, Rosemont, IL, USA, 17435-1-AP), HO1 (Proteintech, 10701-1-AP), 4-hydroxynonenal (4HNE, Abcam, ab46545), acyl-CoA synthetase-4 (ACSL4, Santa Cruz, CA, USA, SC-365230) and β-actin (Beyotime, AF003). Primary antibodies, along with HRP-conjugated secondary goat anti-rabbit and goat anti-mouse antibodies (Beyotime, A0216 and A0208), were diluted following the manufacturer’s guidelines. Image J software (Image J v1.46a; NIH, Bethesda, MD, USA) was used to quantify the densitometry of protein bands.

### 2.10. Measurement of Mitochondrial Membrane Potential

In 6-well plates, 1 × 10^5^ cells per well were incubated in a humidified incubator containing 5% CO_2_ at 37 °C. After treatment, the intracellular distribution and membrane potential (ΔΨm) of mitochondria in GC-1spg cells were labeled with 200 nM Mito-Tracker Red CMXRos (Beyotime) in culture media at 37 °C for 20 min. Mitochondria were visualized by fluorescence microscopy according to the manufacturer’s instructions (Leica, DHI300B).

### 2.11. Assessment of ROS

Superoxide anion levels were detected using dihydroethidium (DHE). Frozen sections (10 μm) were washed twice in PBS, then loaded with DHE (5 μM) at 37 °C in the dark for 30 min. Images were captured using a fluorescence microscope.

As for GC-1 spg cells, cells were cultured in 6-well plates (1 × 10^5^ cells per well). The cells were exposed to drugs for 24 h, then incubated with 5 μM DHE in a humidified incubator with 5% CO_2_ at 37 °C for 30 min in the dark, followed by three washes with PBS. Cells were viewed under a fluorescent microscope.

### 2.12. Statistical Analysis

All data are expressed as the mean ± standard error of the mean. Comparisons between two groups were performed using the unpaired two-tailed Student’s *t*-test, while multiple comparisons were performed using one-way analysis of variance. In this study, a *p* value below 0.05 was deemed statistically significant, and all data were visualized using GraphPad Prism 8 software (San Diego, CA, USA).

## 3. Results

### 3.1. Exposure to BU Induces Testicular Injury

**Figure 1:**
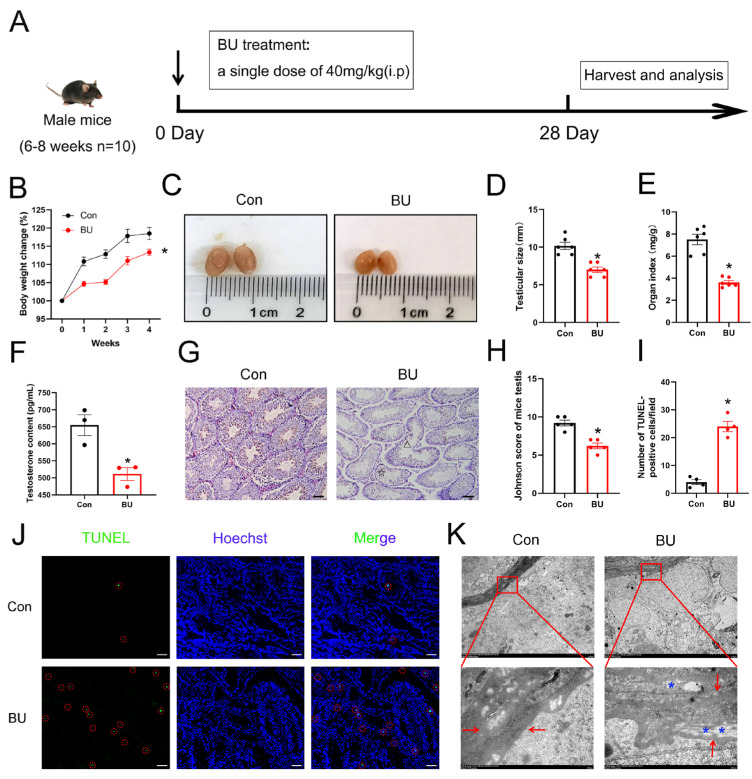
Effects of BU on the testicular structure and function of mice. (**A**) Animal experiment design. Mice were randomly assigned to the following groups and treated for 28 days as follows: control, saline (10 mL/kg i.p.); and BU (40 mg/kg i.p.). (**B**) Changes in the body weight of mice in each group during the experiment. (**C**) Macroscopic changes of gross pathology. (**D**) The testis volume of mice (**E**) Effects of the BU on organ indices. (**F**) Serum testosterone content. (**G**,**H**) H&E staining and Johnson scores of mouse testes (scale bars 100 μm). (Δ) disorganization of seminiferous tubules, (**☆**) exfoliated germ cells in the tubular lumen. (**I**,**J**) TUNEL staining of testicular sections (scale bars: 100 μm) and quantification of apoptotic cells. Red circle: Apoptotic cells. (**K**) Changes in the basement membrane structure of mice, (**⟵**) basement membrane, (*) vacuolization of basement membrane (scale bars 5 μm and 500 nm). Data are presented as mean ± SEM. * *p* < 0.05 vs. the control group.

Initially, we created a mouse model to study testicular damage caused by BU, focusing on its toxic impact on spermatogonia. As shown in Figure 1A, C57BL/6J mice were treated with different drugs. After 28 days of treatment, BU-exposed mice exhibited significantly slower body weight gain despite equivalent initial body weights, reflecting the poor health status of the mice (Figure 1B). Testicular atrophy and testicular shrinkage were also noted in BU-treated mice (Figure 1C). In addition, the testis volume and the testicular organ coefficient of the BU group were significantly decreased compared to those of the control group (Figure 1D,E). Next, we measured serum testosterone levels and found that testosterone levels were increased following administration of BU (Figure 1F). H&E staining revealed that the germ cells in BU-treated mice were disordered within seminiferous tubules and seminiferous tubule lumens were enlarged compared to untreated mice (Figure 1G,H). TUNEL staining combined with quantitative analysis revealed that BU treatment significantly increased apoptotic cells in the testes compared to the control group (Figure 1I,J). Ultrastructural analysis revealed BU-induced pathological changes in the seminiferous tubule basement membranes, such as thickening, surface irregularities (→, Figure 1K), and vacuolization (*, Figure 1K). Together, our results showed that the chemotherapeutic drug BU affected the reproductive function of male mice.

### 3.2. BU Exposure Leads to Ferroptosis in Mouse Testes

**Figure 2:**
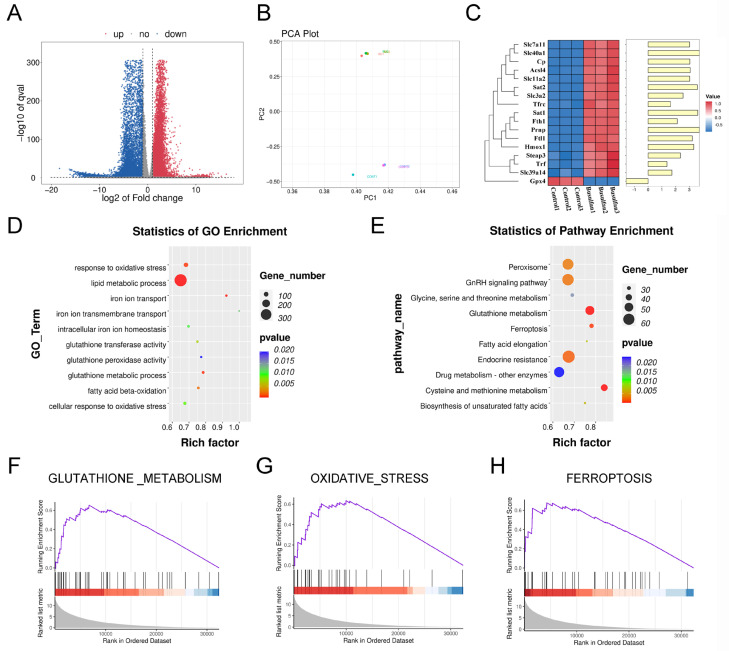
BU promotes GSH metabolism disorders and induces ferroptosis. (**A**) Volcano plot showing that a total of 18,477 DEGs were obtained from RNA-seq data of mouse testes, including 8973 upregulated DEGs and 9504 downregulated DEGs. (**B**) PCA showing good intergroup consistency in testicular cells. (**C**) Heat map showing relative mRNA expression levels of ferroptotic genes in the mouse testes. (**D**) GO analysis based on RNA-seq data of mouse testes showing enrichment of DEGs in oxidative stress- and iron metabolism-related GO terms. (**E**) KEGG analysis based on RNA-seq data results of mouse testes after exposure to BU. (**F**) GSEA analysis showing activation of GSH metabolism based on RNA-seq data of mouse testes. (**G**) GSEA analysis showing activation of oxidative stress based on RNA-seq data of mouse testes. (**H**) GSEA analysis showing enhanced ferroptosis signaling pathway based on RNA-seq data of mouse testes. n = 3.

To explore the mechanisms behind BU-induced testicular damage, we conducted RNA-seq to identify DEGs between the control and BU groups. Figure 2A outlines our RNA-seq data analysis for testes. Principal component analysis (PCA) showed good intergroup consistency in cells (Figure 2B). The results showed 8973 upregulated DEGs and 9504 downregulated DEGs (Figure S1A, Table S2). The heat map from ferroptotic DEGs is shown in Figure 2C. GO analysis indicated that DEGs were concentrated in GO terms related to oxidative stress and iron metabolism (Figure 2D). KEGG analysis showed that DEGs were concentrated in the ferroptosis pathway (Figure 2E). In addition, GSEA indicated that the oxidative stress and GSH metabolic pathways were enhanced, and the ferroptotic pathway was upregulated (Figure 2F–H). Upregulation of ferroptotic genes including *Tfr*, *Fth1*, ferroportin (*Fpn*), and *Acsl4* was also observed. However, *Gpx4* was shown to be downregulated after BU exposure (Figure 2C).

**Figure 3:**
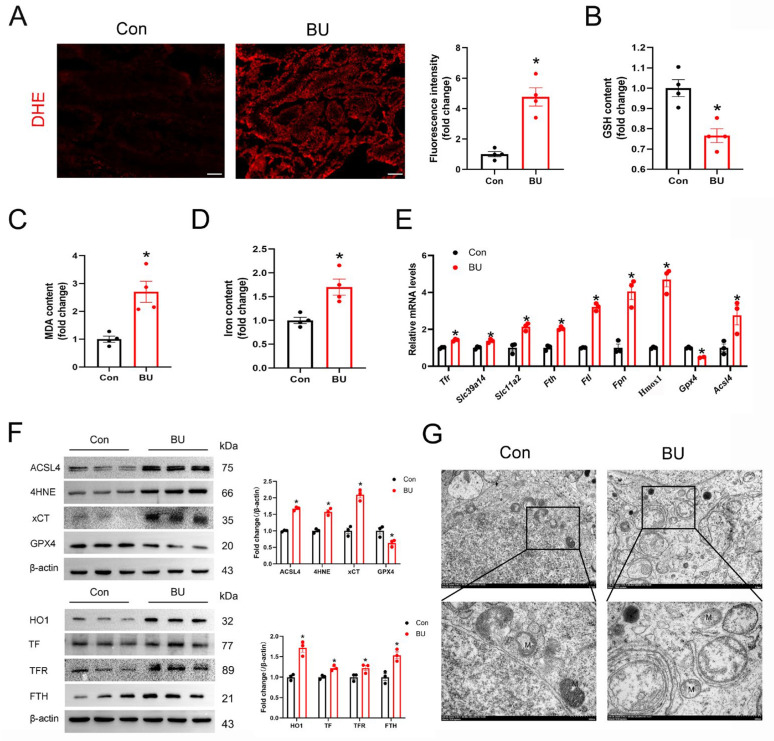
BU exposure induces ferroptosis in mouse testes. (**A**) Representative images showing DHE staining in testicular tissue sections (scale bars 100 μm). (**B**,**C**) GSH and MDA content in mouse testes (n = 4). (**D**) Testicular iron content (n = 4). (**E**) RT-qPCR analysis showing ferroptotic-related mRNA levels. (**F**) Representative western blot and semi-quantitative analysis of ferroptotic protein expression levels in mice. (**G**) Representative transmission electron microscopic images showing the testes of different treatment groups. M, mitochondria (scale bars 2 μm and 500 nm). Data are presented as mean ± SEM. * *p* < 0.05 vs. the control group.

To verify our previous RNA-seq results for testes, we have also conducted relevant animal experiments. Because the accumulation of lipid peroxides and iron accumulation are characteristic of ferroptosis, we first assessed indices of ferroptosis, including ROS products (Figure 3A). We found that BU-induced MDA production and GSH depletion in the testes (Figure 3B,C). Furthermore, a significant accumulation of iron was observed in the testes of the BU group (Figure 3D). Our qPCR analysis showed upregulation of *Tfr*, solute carrier family 39 member 14 (*Zip14*), solute carrier family 11 member 2 (*Dmt1*), *Fth1*, ferritin light chain (*Ftl*), *Fpn*, *Hmox1, Slc7a11* and *Acsl4* expression levels, together with downregulation of *Gpx4* (Figure 3E). In addition, our western blotting data showed upregulation of 4HNE, a marker of ferroptosis, in the BU group (Figure 3F and Figure S3). BU exposure resulted in abnormal mitochondrial morphology was abnormal with fewer mitochondrial cristae and denser outer membranes in mouse testes (Figure 3G). Together, these findings indicated that BU exposure induced ferroptosis in the testes.

### 3.3. BU Reduces Cell Viability and Induces Ferroptosis in GC-1 Spg Cells

**Figure 4:**
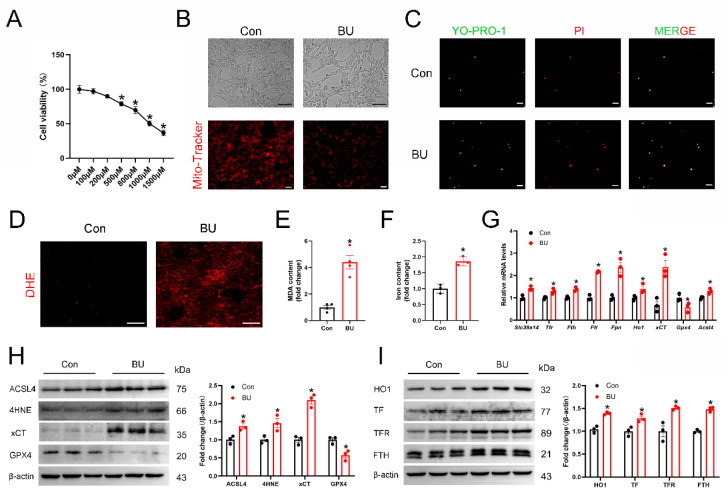
BU inhibits viability and induces ferroptosis in GC-1 spg cells. (**A**) CCK-8 assay showing that BU treatment leads to a dose-dependent reduction in the viability of GC-1spg cells. (**B**) Morphology and mitochondrial membrane potential of GC-1 spg cells following treatment with BU (800 μM) for 24 h (scale bars 100 μm and 20 μm). (**C**) Representative fluorescence images showing YP1/IP staining of GC-1 spg cells (scale bars 20 μm). (**D**) Representative images showing ROS levels in GC-1 spg cells (scale bars 20 μm). (**E**,**F**) MDA levels and iron content in GC-1 spg cells. (**G**) qRT-PCR analysis of mRNA levels of ferroptotic genes. (**H**,**I**) Western blot analysis of ferroptotic proteins in GC-1spg cells (n = 3). Data are presented as the mean ± SEM. * *p* < 0.05 vs. the control group.

According to research, the main target cells of BU-causing male reproductive problems are spermatogonia. To adhere to the toxicological research on BU’s effects on the testes, pertinent experiments were performed in vitro using a spermatogonial cell line (GC-1 spg cells). To further investigate the role of BU in male reproductive function, the GC-1 spg cells were pretreated with BU. In vitro, BU treatment led to a dose-dependent reduction in GC-1 spg cell viability as measured by the CCK8 assay (Figure 4A), and visualized by light microscopy (Figure 4B). Based on these results, BU was used at a concentration of 800 μM in subsequent experiments. Using a mitochondrial fluorescent probe, we demonstrated that BU treatment altered mitochondrial morphology and reduced the mitochondrial membrane potential of GC-1 spg cells (Figure 4C). Next, cell viability was assessed by YPI/PI staining. We found a significantly higher level of cell death in BU-treated cells than untreated cells. Increased cellular ROS levels (Figure 4D), together with increased MDA levels (Figure 4E), and iron accumulation (Figure 4F) were also observed after BU exposure. Moreover, the changes in ferroptosis-related gene and protein expression levels following BU treatment were consistent with our in vivo data (Figure 4G–I and Figure S4). Together, our results demonstrated that BU-induced accumulation of iron and lipid peroxides in GC-1 spg cells via activation of ferroptosis.

### 3.4. Ferroptosis Regulates BU-Induced GC-1 Spg Cell Death

**Figure 5:**
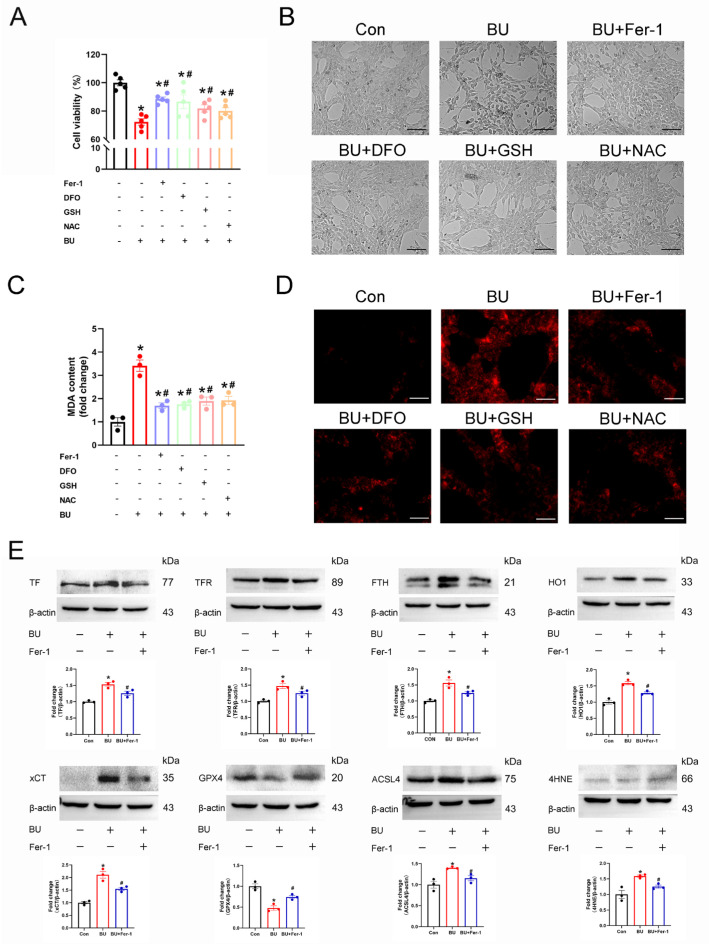
Ferroptosis regulates BU-induced GC-1spg cell death. (**A**) CCK-8 assay showing that BU-induced GC-1 spg cell death was mitigated in the presence of Fer-1 (1 μmol/L), DFO (50 μmol/L), GSH (5 mmol/L) or NAC (5 mmol/L). (**B**) Representative light microscopic images of GC-1spg cells and the changes in ROS fluorescence intensity after treatment of L-02 cells with Fer-1, DFO, GSH, or NAC (scale bars 100 μm). (**C**) BU-induced increases in lipid peroxidation were reversed in the presence of ferroptosis inhibitors in GC-1 spg cells. (**D**) Representative images showing ROS fluorescence intensity after treatment of GC-1spg cells with Fer-1, DFO, GSH, or NAC (scale bars 100 μm). (**E**) Differential expression of ferroptotic proteins in GC-1 spg cells was following Fer-1 treatment. Data are presented as mean ± SEM (n = 3). * *p* < 0.05 vs. the control group. # *p* < 0.05 vs. the BU group.

To further investigate the role of ferroptosis in male reproductive diseases induced by BU, the GC-1 spg cells were pretreated with various inhibitors of ferroptosis. BU exposure significantly reduced the viability of GC-1 spg cells. However, reduced cell viability was ameliorated with the use of an iron chelator (DFO), a ferroptosis inhibitor (Fer-1), and the antioxidants that scavenge ROS inside the cell (GSH and NAC) (Figure 5A,B). Similarly, BU-induced increases in cellular ROS and MDA levels in GC-1spg cells were rescued following treatment with ferroptosis inhibitors (Figure 5C,D). Furthermore, western blotting analysis showed that Fer-1 (a ferroptosis-specific inhibitor) restored the upregulation of TF, TFR, FTH, xCT, HO1, ACSL4, and 4HNE expression levels, and downregulation of GPX4 after BU exposure (Figure 5E and Figure S5). Together, our results showed that ferroptosis regulated BU-induced GC-1 spg cell death.

### 3.5. The HO1 Inhibitor ZnPP Suppresses BU-Induced Ferroptosis in GC-1 Spg Cells

**Figure 6:**
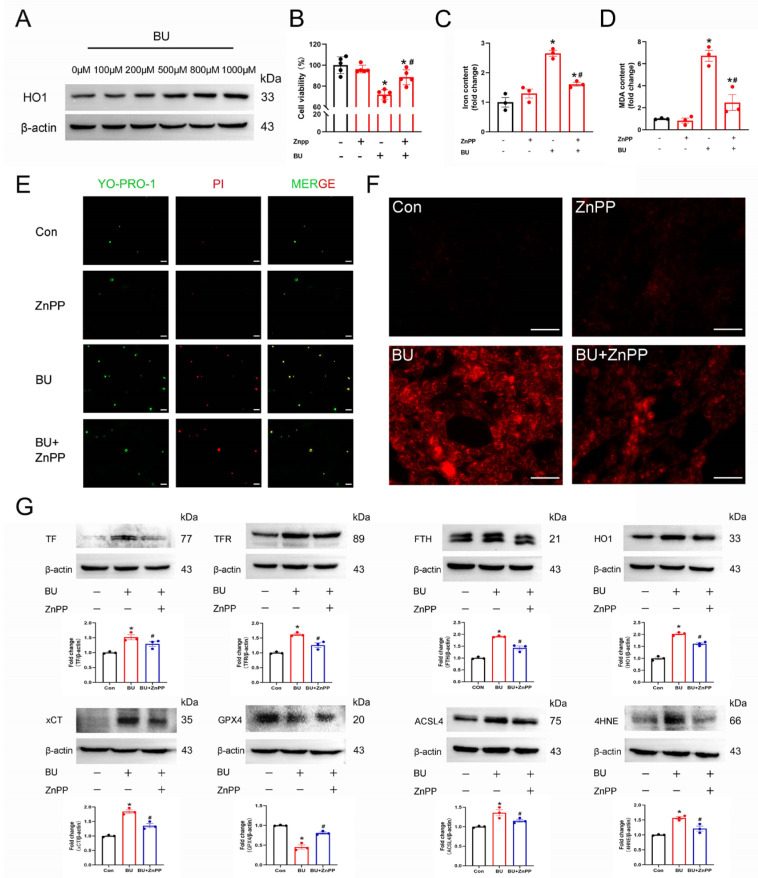
BU induces ferroptosis via the HO1 pathway in GC-1 spg cells. (**A**) Western blot analysis showing that BU treatment led to a dose-dependent increase in HO1 expression levels. (**B**) CCK-8 assay showing that BU-induced GC-1 spg cell death was mitigated by pretreatment with ZnPP (1 μM). (**C**) Pretreatment with DFO mitigates BU-induced increases in intracellular iron levels in GC-1 spg cells. (**D**) BU-induced increases in lipid peroxidation are reversed in GC-1 spg cells pretreated with ZnPP. (**E**) The effects of ZnPP pretreatment on BU-induced cell death were analyzed by YP1/IP staining (scale bars 20 μm). (**F**) Changes in ROS fluorescence intensity were detected in GC-1 spg cells pretreated with ZnPP (1 μM) for 1 h followed by treatment with BU (800 μM) for 24 h (scale bars 100 μm). (**G**) ZnPP treatment reverses the differential expression of ferroptotic proteins in GC-1 spg cells. * *p* < 0.05 vs. the control group. # *p* < 0.05 vs. the BU group.

Increasing evidence has shown that HO1 plays a critical role in ferroptosis, with high HO1 expression levels associated with cytoprotective effects in various stress-related conditions. Western blotting revealed that HO1 protein expression levels were significantly increased in a dose-dependent manner following treatment of GC-1 spg cells with BU for 24 h (Figure 6A and Figure S6), while the expression of NRF2 and GPX4 were downregulated (Figures S2 and S7). However, pretreatment with ZnPP (1 μΜ) for 1 h effectively reduced the levels of cell death as measured by the CCK8 assay (Figure 6B). BU-induced increases in MDA levels (Figure 6C), and iron accumulation (Figure 6D) in GC-1 spg cells were also rescued following pretreatment with ZnPP. Increased MDA (Figure 6C), and iron accumulation (Figure 6D) after BU exposure were also rescued using ZnPP in GC-1spg cells. Similarly, YO-PRO-1/PI staining demonstrated that pretreatment with ferroptosis inhibitors rescued BU-induced cell death (Figure 6E). Significantly increased DHE staining was observed in BU-treated cells, while ZnPP attenuated this trend (Figure 6F). Western blot analysis revealed that Fer-1 treatment rescued the abnormal expression of ferroptosis-related proteins induced by BU (Figure 6G and Figure S6). Together, our findings showed that HO1 mediated BU-induced ferroptosis in GC-1 spg cells.

## 4. Discussion

**Figure 7:**
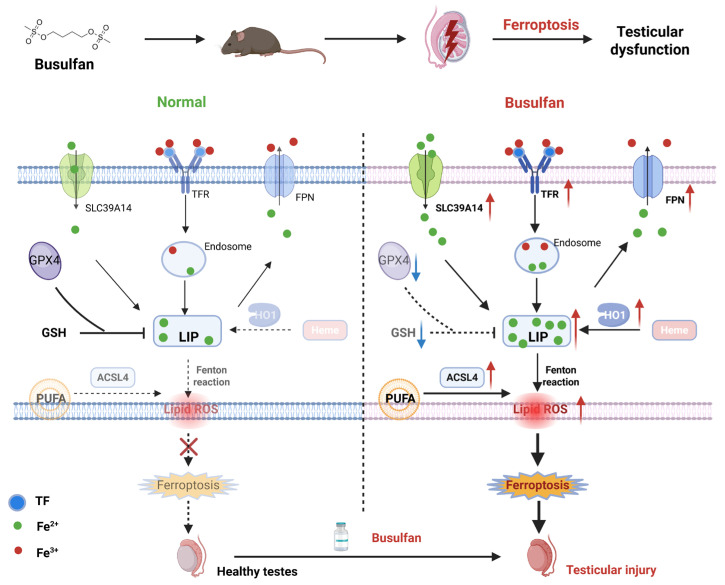
Schematic diagram showing BU-induced testicular injury in mice.

BU, a key drug in first-line treatments, works well with other agents but causes testicular damage due to its breakdown in the testes. This highlights the need for strategies that balance treatment effectiveness with protecting reproductive health [23,30,31]. Thus, understanding the molecular mechanisms of BU-induced reproductive toxicity is critical. In the current study, BU treatment of mice led to significant weight loss and decreased activity 28 days after treatment. The main results are described as follows. BU-exposed mice displayed significant hypoactivity with reduced spontaneous movement and exploratory behavior, accompanied by neurobehavioral depression manifested through prolonged immobility, delayed responses to auditory/tactile stimuli, and diminished nest-building capacity. Treated animals exhibited progressive coat deterioration characterized by rough, lusterless fur texture and localized piloerection, along with marked growth retardation evidenced by 20–30% reduced body weight gain rates, decreased food/water consumption, and delayed developmental milestones compared to controls. Biochemical indexes showed that BU decreased the levels of testosterone in the serum of mice. In addition, BU exposure led to testicular injury, accompanied by excessive ROS accumulation and increased tissue iron content. Our in vitro studies showed that BU treatment led to a reduction in the viability of GC-1 spg cells, which was accompanied by an abnormal increase in intracellular iron content and lipid peroxide levels. At the same time, we found that the inhibition of lipid peroxidation and mitochondrial dysfunction with Fer-1, as well as GSH and NAC could also rescue BU-induced cell death. Similarly, treatment with the iron chelator DFO also inhibited BU-induced cell death. Our findings showed that ferroptosis mediates BU-induced testicular injury. In addition, we confirmed that BU not only caused ferroptosis through excessive iron intake, but also further aggravated iron content in tissues through the HO1 pathway (Figure 7).

Iron is involved in numerous biological reactions such as DNA synthesis and repair, hemoglobin oxygen transport, free radical induction and production, and many other important reactions [32]. Therefore, dysregulation of iron homeostasis can have adverse effects on the body [33]. Disruption of the balance between iron uptake, storage, and export, which leads to an abnormal increase in intracellular ferrous iron (Fe^2+^) levels, is considered to be a critical factor in ferroptosis. Excessive accumulation of Fe^2+^ in cells is the direct cause of ferroptosis. The TF-TFR and ZIP14 pathways mediate the ingestion of extracellular TF-bound and non-TF-bound iron into cells, while the ferroportin (FPN) in the cytoplasmic membrane exports Fe^2+^ from cells [34,35]. Intracellular Fe^2+^ that is not immediately used for cellular processes is stored by ferritin, which consists of heavy (FTH) and light (FTL) chains. In the current study, we consistently showed that BU significantly upregulated the expression of proteins or genes associated with iron intake, which may explain the abnormal increase in iron content in testicular tissue induced by BU. In addition to excessive iron intake, impaired iron output, also known as ferritinophagy, can lead to iron accumulation [36]. Here, we found that BU increased intracellular iron levels by upregulating TF-TFR, which transports iron through the blood, as well as the iron transporter ZIP14. BU did not increase intracellular iron levels through the inhibition of ferritin or ferroportin. Furthermore, our findings suggested that the BU-induced increase in FTH, FTL, and FPN was an adaptive stress response to the BU-induced increase in intracellular iron.

The xCT and GPX4 proteins play a critical role in protecting cells from oxidative stress damage [37]. The xCT accounts for the transport of extracellular cystine into the cell, which is then reduced to cysteine for GSH synthesis. GPX4 can catalyze the reduction of lipid peroxides by depleting GSH as a reduction cofactor [38]. The xCT/GPX4 pathway plays an important role in protecting cells from ferroptosis [37,39]. Supplementation with GSH or NAC inhibited BU-induced GC-1 spg cell death, as well as overproduction of ROS and lipid peroxides. Moreover, xCT expression was found to be significantly increased in both in vitro and in vivo experiments. Our data indicated that BU induced the downregulation of GPX4 both in vitro and in vivo. Thus, BU may induce ferroptosis via inhibition of GPX4.

It is worth noting that HO1 plays a dual role in the regulation of ferroptosis. HO1 oxidizes cellular heme to carbon monoxide, Fe^2+^, and biliverdin. On the one hand, under normal conditions, high expression of HO1 helps to attenuate oxidative stress and exert a cytoprotective effect. On the other hand, sustained HO1 upregulation releases Fe^2+^ to exacerbate oxidative stress, leading to ROS accumulation through the Fenton reaction, particularly when NRF2 and GPX4 are inactive [40,41]. The role of HO1 in the regulation of ferroptosis has been reported in different pathological models, such as liver injury, testicular injury, and colitis mouse models [42,43]. In our study, we demonstrated that ferroptosis occurred during BU-induced testicular injury, and that BU-induced upregulation of HO1 in a dose-dependent manner. ZnPP, a HO1 inhibitor, reversed the effects of BU on ferroptosis. Therefore, our findings suggest that elevated HO1 may be a cause of ferroptosis. Upregulation of HO1 due to BU exposure may exacerbate oxidative stress and lead to excessive release of Fe^2+^ from the testes in mice. Our findings highlight the clinical potential of combining ferroptosis inhibitors with BU to mitigate testicular toxicity. First, we demonstrated that ZnPP, a HO1 inhibitor, effectively rescued BU-induced ferroptosis in testicular cells. HO1 exacerbates iron accumulation by degrading heme to release free iron, thereby fueling lipid peroxidation—a hallmark of ferroptosis in iron overload disorders such as hemochromatosis [44]. Studies have shown that inhibiting HO-1 significantly enhances the effects of gemcitabine and radiotherapy, emphasizing that HO-1 inhibition can serve as a potential strategy to improve pancreatic cancer treatment outcomes and provide new ideas for clinical practice [45], suggesting their utility as adjuvant agents in BU-based regimens. Second, iron chelators like DFO may offer synergistic protection by reducing the labile iron pool (LIP) and suppressing Fenton reaction-driven lipid peroxidation. Notably, DFO has been shown to attenuate cisplatin-induced nephrotoxicity and ifosfamide-associated ferroptosis while preserving chemotherapy efficacy [46,47]. Together, targeting HO1 and iron metabolism represents a dual-pronged approach to decouple BU’s therapeutic effects from its gonado toxicity, warranting further validation in translational models.

Furthermore, the molecular regulatory relationship between BU and ferroptosis has not been elucidated yet, and further research is needed to further clarify it. Finally, we performed our experiments in spermatogonial cell lines because previous experiments have shown that BU can extensively deplete spermatogonia. Some studies have suggested that exposure to busulfan impairs the structural integrity of Leydig cells, leading to reduced testosterone levels and subsequent disruptions in spermatogenesis [48]. Furthermore, busulfan-induced testicular dysfunction is not limited to Leydig cells. It also affects Sertoli cells, which are crucial for supporting spermatogenesis. Increased expression of spermidine/spermine N1-acetyltransferase 2 (Sat2) in Sertoli cells has been associated with busulfan treatment, leading to inhibited cell proliferation and arrested cell cycle. This disruption in Sertoli cell function further exacerbates the negative impact on spermatogenesis, contributing to male infertility [49].

However, there were several limitations in the study. First, this study aims to explore the mechanism of ferroptosis in testicular injury induced by BU, so we did not delve into other possible related pathways and key regulatory molecules, although we conducted the RNA sequencing. As shown in Figure S1, RNA-seq results predict other possible mechanisms of BU-induced testicular injury, such as tight junction, DNA synthesis, apoptosis, and necrosis. This highlights the complexity of the mechanism of BU-induced testicular injury and requires further experimental clarification. Recently, it was shown that BU, as a DNA alkylating agent, likely activates ferroptosis via DNA damage response (DDR)-mediated mechanisms: (1) DDR kinases (ATM/ATR) disrupt iron homeostasis by upregulating transferrin receptor and suppressing ferritinophagy, elevating labile iron to fuel lipid peroxidation; (2) p53, activated by persistent DNA breaks, transcriptionally represses SLC7A11, depleting glutathione and disabling GPX4 antioxidant defense. (3) These pathways synergize with mitochondrial dysfunction, creating a self-reinforcing loop that commits testicular cells to ferroptosis [50,51]. Further studies are required to evaluate the impact of DNA fragmentation patterns (as quantified by SCD assays) on iron homeostasis dysregulation.

BU-induced ROS accumulation plays dual roles in testicular injury pathogenesis, driving lipid peroxidation while activating adaptive metabolic pathways. Oxidative cysteine modifications (e.g., modification of Cys215 on Insig-2) suppress lipid synthesis through SREBP signaling and redirect metabolic flux toward alternative energy pathways [52], whereas ROS-mediated BAK/BAX activation and mitochondrial permeabilization enhance apoptotic-ferroptotic interplay [53]. Concurrently, redox-sensitive N-degron pathways modulate protein homeostasis under oxidative stress conditions [54]. These interconnected mechanisms position ROS as dual-function mediators in germ cell survival-serving as cytotoxic effectors and metabolic regulators-underscoring the need to investigate oxidative cysteine-based post-translational modifications in ferroptosis regulation.

## 5. Conclusions

In summary, we systematically demonstrated that ferroptosis mediated BU-induced testicular injury. BU increased intracellular iron levels by upregulating the expression of iron intake-related genes and proteins, which inhibited GPX4 expression. In addition, BU further exacerbated iron accumulation by upregulating HO1 levels, depleting intracellular GSH levels, and inducing oxidative stress, which further enhanced intracellular lipid peroxidation levels. Our findings provided novel insights to support further investigation of BU toxicity to better understand the mechanisms of male reproductive toxicity.

## Data Availability

The data presented in the manuscript are not deposited in an official repository but can be made available upon reasonable request.

## Supplementary Materials

The following supporting information can be downloaded at https://www.mdpi.com/article/10.3390/biology14060594/s1, Figure S1: RNA-seq in mouse testes after exposure to BU; Figure S2: BU induced the oxidative stress of testes; Figure S3: The original Western blot images for Figure 3; Figure S4: The original Western blot images for Figure 4; Figure S5: The original Western blot images for Figure 5; Figure S6: The original Western blot images for Figure 6; Figure S7: The original Western blot images for Figure S2; Table S1: Primer sequences. Table S2: Upregulated and downregulated differentially expressed genes.

## Abbreviations

ACSL4	acyl-CoA synthetase long-chain family member 4
BU	Busulfan
DFO	Desferrioxamine
DHE	Dihydroergotamine
DMT1/SLC11A2	Solute carrier family 11 member 2
Fer-1	Ferrostatin-1
FPN	Ferroportin
FTH	Ferritin, heavy polypeptide
FTL	Ferritin, light polypeptide
GPX4	Glutathione peroxidase 4
GSH	Glutathione
HO1	Heme oxygenase 1
LIP	Labile iron pool
MDA	Malondialdehyde
NAC	N-Acetyl-L-cysteine
NRF2	Nuclear factor erythroid 2-related factor 2
ROS	Reactive oxygen species
TF	Transferrin
TFR	Transferrin receptor1
TUNEL	Terminal deoxynucleotidyl transferase dUTP nick end labeling
xCT/SLC7A11	Solute carrier family 7 member 11
YO-PRO-1 (YP1)/PI	Oxazole yellow/Propidium Iodide
ZIP14/SLC39A14	Solute carrier family 39 member 14
4HNE	4-Hydroxynonenal
