# Supplementary figures and images for: Ferroptotic Pathway Activation in Spermatogonia: A Novel Mechanism of Busulfan-Induced Testicular Injury
Source: Biology (Basel). 2025 May 23;14(6):594. doi: 10.3390/biology14060594 (PMC12189493; doi:10.3390/biology14060594)

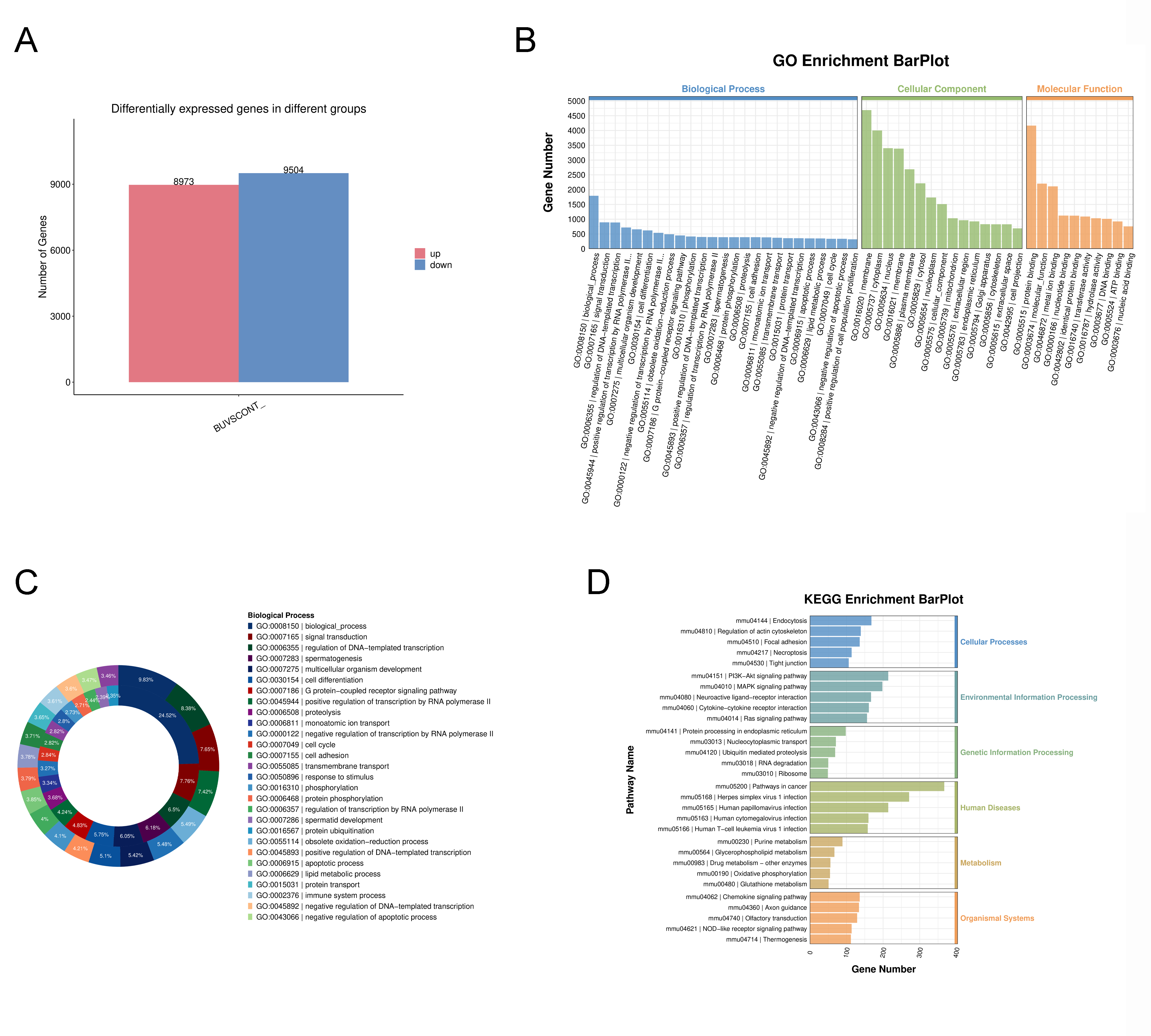

Supplement: Supplementary file 1 [file biology-14-00594-s001.zip › biology-3610255-supplementary/biology-3610255-supplementary/Fig-S1.tif]

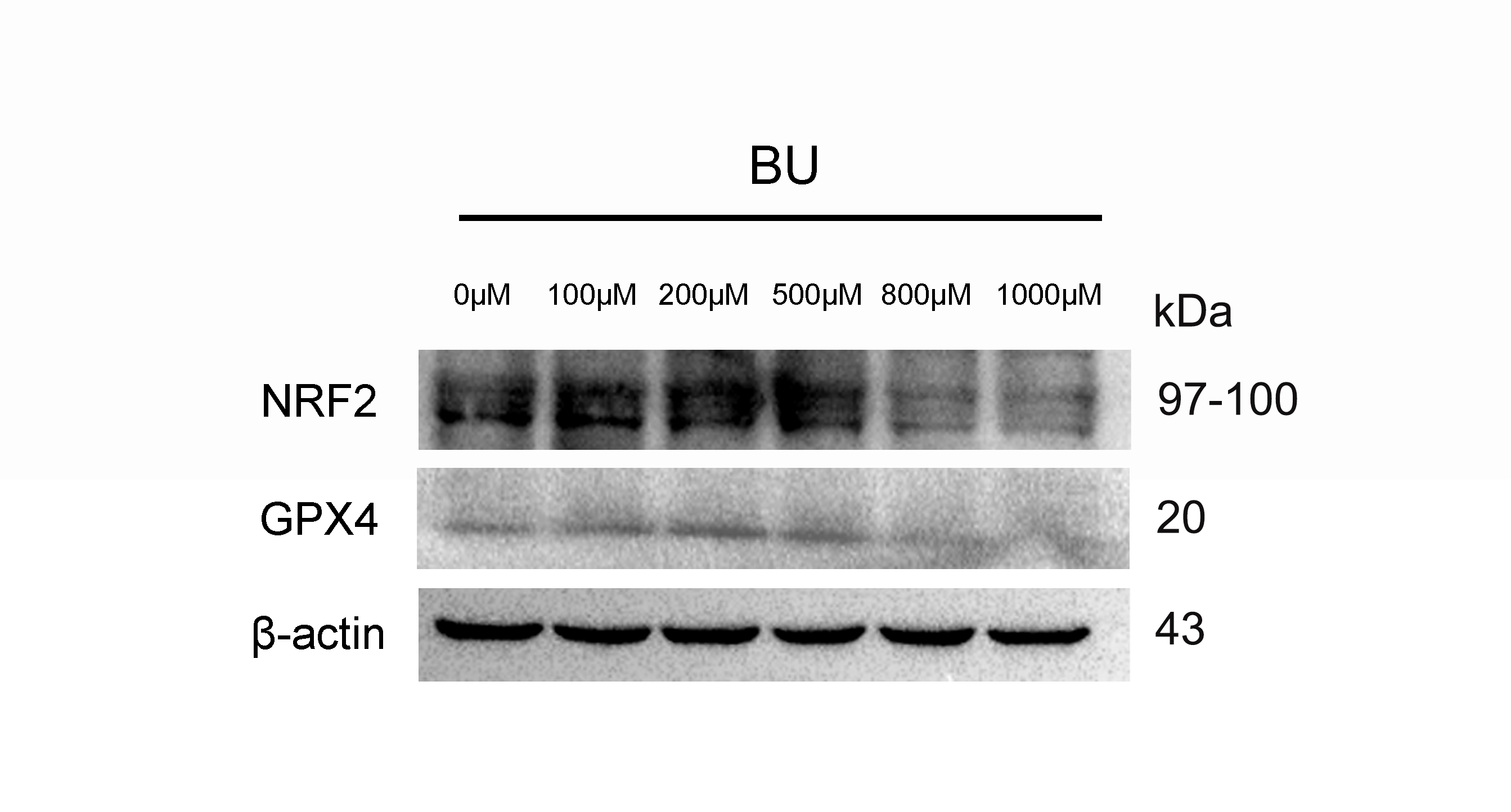

Supplement: Supplementary file 1 [file biology-14-00594-s001.zip › biology-3610255-supplementary/biology-3610255-supplementary/Fig-S2.tif]

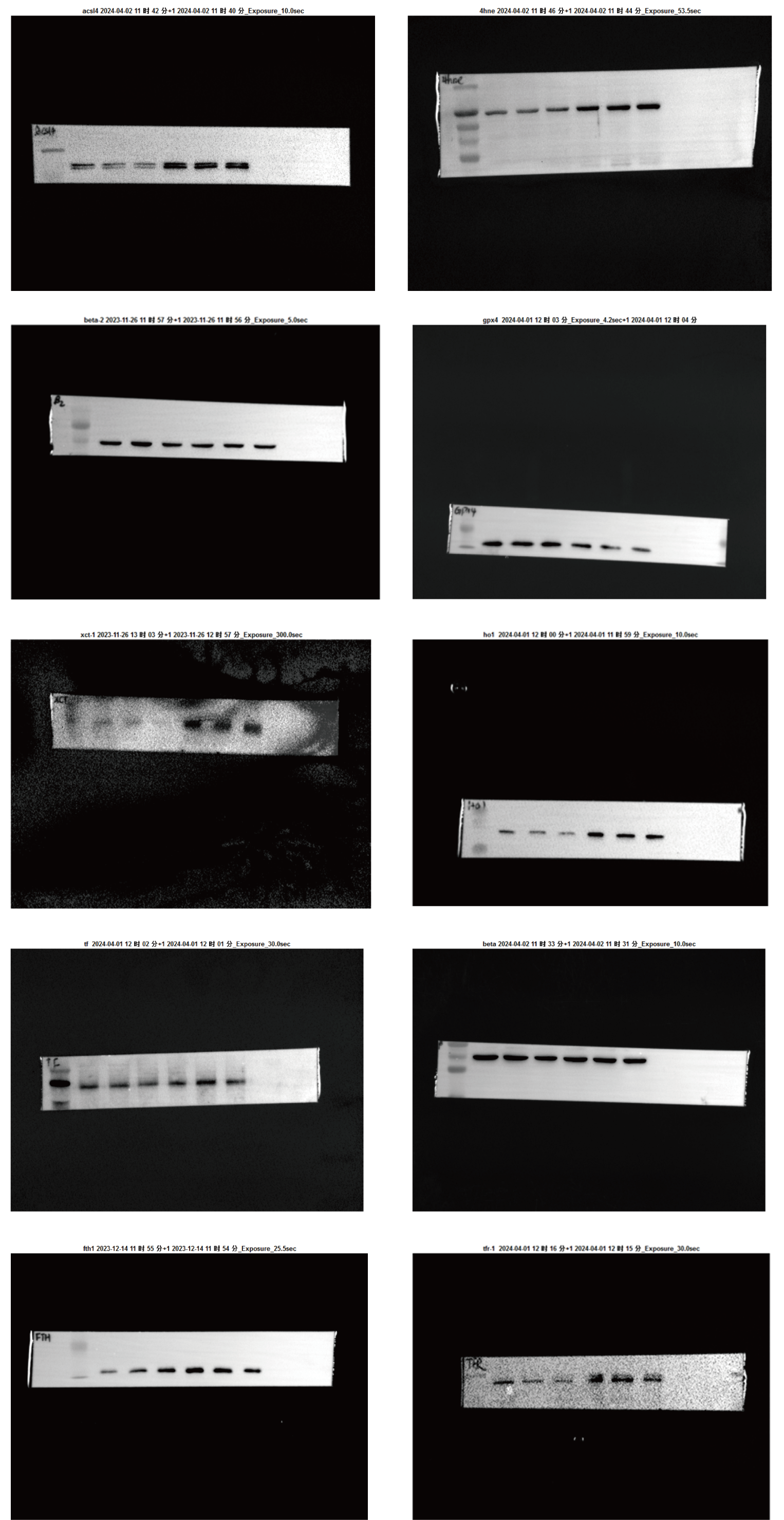

Supplement: Supplementary file 1 [file biology-14-00594-s001.zip › biology-3610255-supplementary/biology-3610255-supplementary/Figure S3.tif]

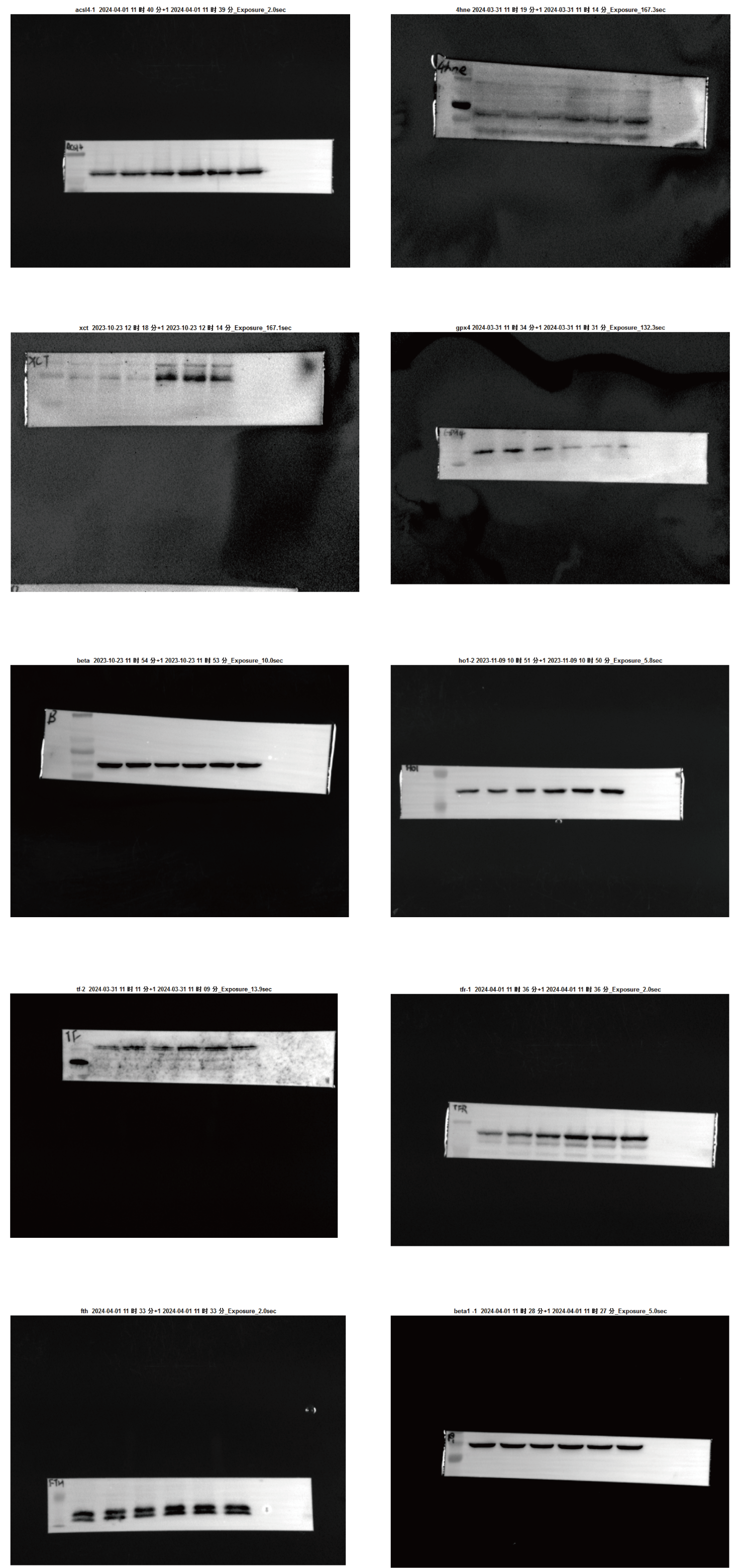

Supplement: Supplementary file 1 [file biology-14-00594-s001.zip › biology-3610255-supplementary/biology-3610255-supplementary/Figure S4.tif]

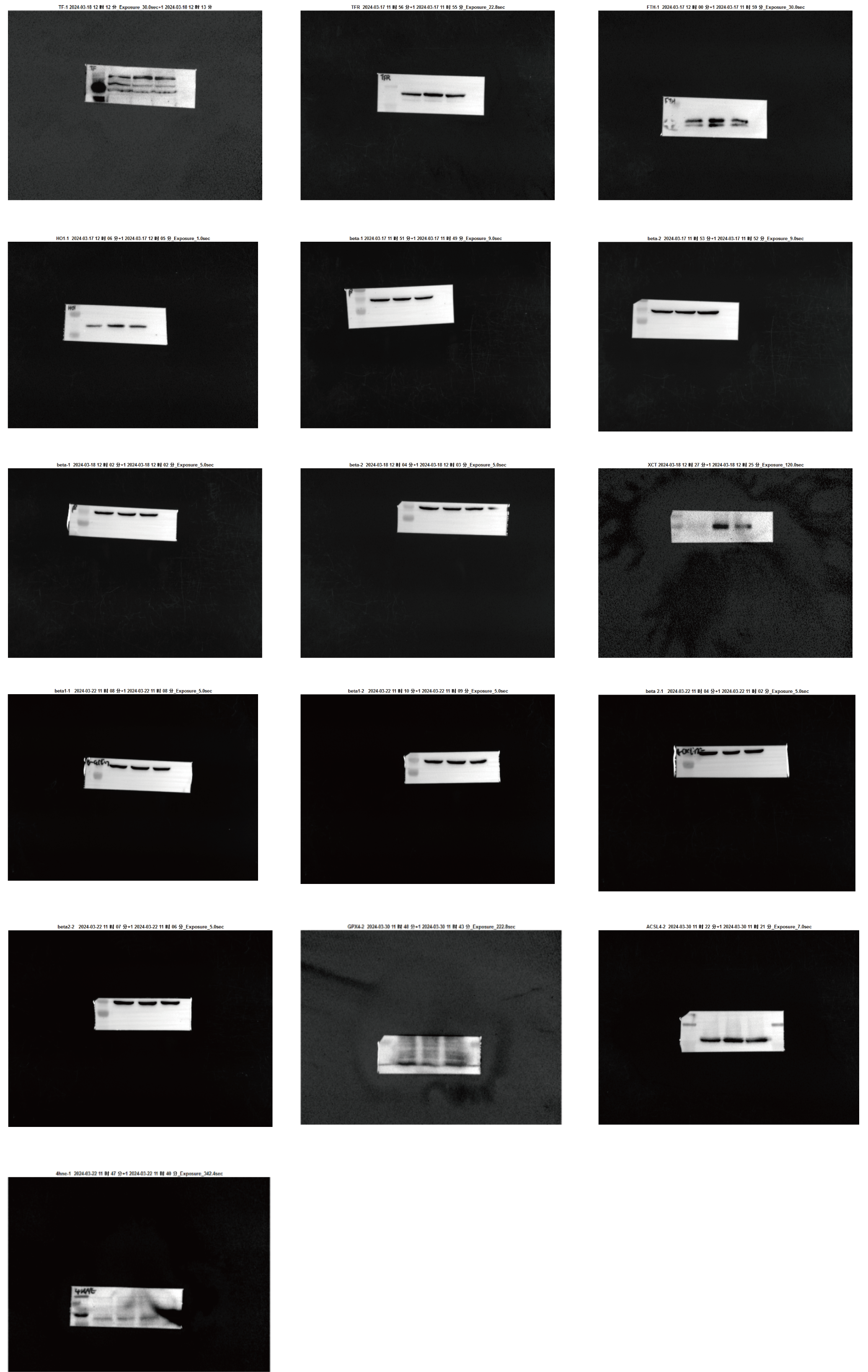

Supplement: Supplementary file 1 [file biology-14-00594-s001.zip › biology-3610255-supplementary/biology-3610255-supplementary/Figure S5.tif]

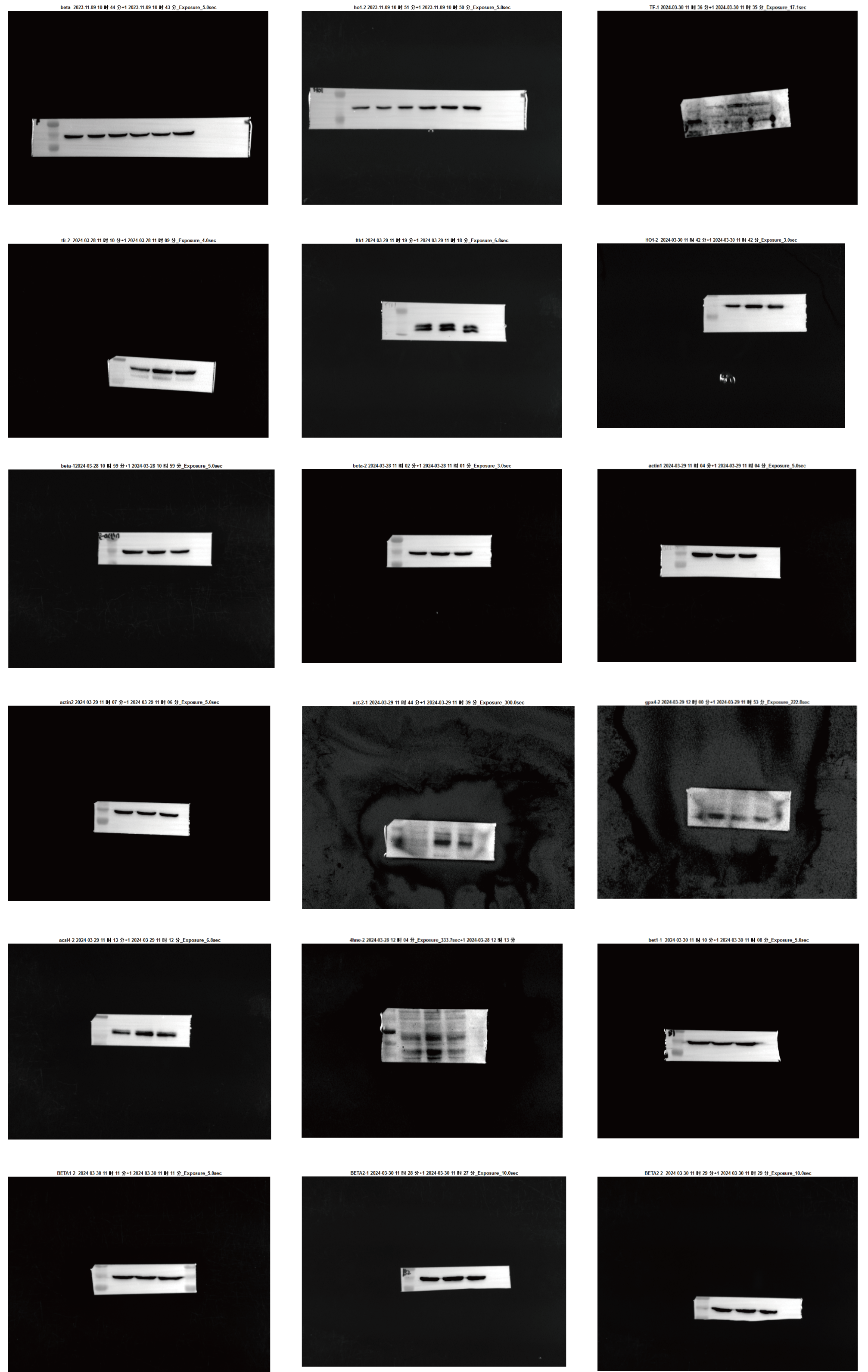

Supplement: Supplementary file 1 [file biology-14-00594-s001.zip › biology-3610255-supplementary/biology-3610255-supplementary/Figure S6.tif]

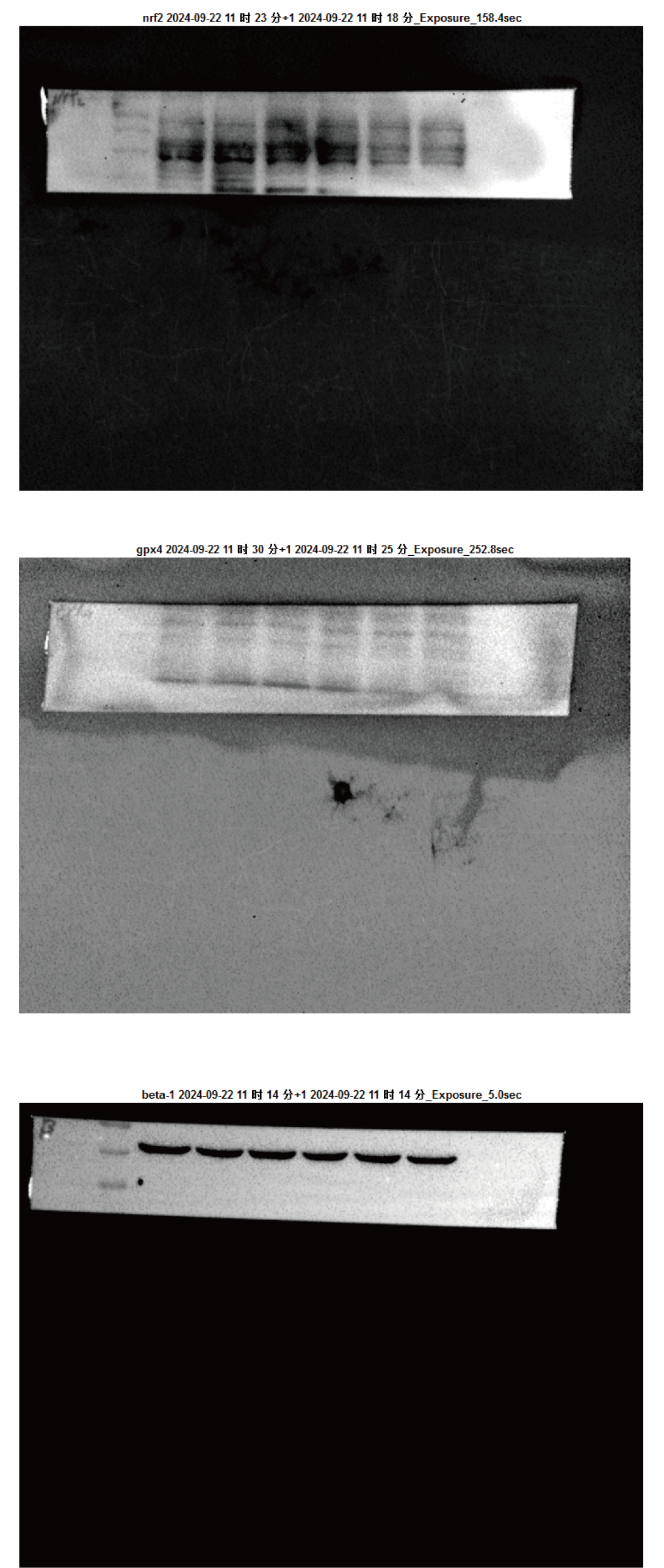

Supplement: Supplementary file 1 [file biology-14-00594-s001.zip › biology-3610255-supplementary/biology-3610255-supplementary/Figure S7.tif]
